# Approaches to pandemic prevention – the chromatin vaccine

**DOI:** 10.3389/fimmu.2023.1324084

**Published:** 2023-12-08

**Authors:** Jielin Zhang, Philip Askenase, Rudolf Jaenisch, Clyde S. Crumpacker

**Affiliations:** ^1^ Department of Medicine, Beth Israel Deaconess Medical Center, Harvard Medical School, Boston, MA, United States; ^2^ Allergy & Clinical Immunology, Yale School of Medicine, New Haven, CT, United States; ^3^ Department of Biology, Whitehead Institute, Massachusetts Institute of Technology, Cambridge, MA, United States

**Keywords:** vaccine efficacy, vaccine breakthrough, viral pathogenicity, chromatin vaccine (cVacc), epigenetic immunity

## Abstract

Developing effective vaccines against viral infections have significant impacts on development, prosperity and well-being of human populations. Thus, successful vaccines such as smallpox and polio vaccines, have promoted global societal well-being. In contrast, ineffective vaccines may fuel arguments that retard scientific progress. We aim to stimulate a multilevel discussion on how to develop effective vaccines against recent and future pandemics by focusing on acquired immunodeficiency syndrome (AIDS), coronavirus disease (COVID) and other viral infections. We appeal to harnessing recent achievements in this field specifically towards a cure for current pandemics and prevention of the next pandemics. Among these, we propose to apply the HIV DNA in chromatin format – an end product of aborted HIV integration in episomal forms, i.e., the chromatin vaccines (cVacc), to elicit the epigenetic silencing and memory that prevent viral replication and infection.

## Introduction

COVID-19 occurred after the AIDS pandemic. The treatment victories against AIDS have provided lessons for COVID-19, and new bioscience approaches against both AIDS and COVID-19 have developed new lessons for developing preventions of the next pandemics.

Recently, in-house pharmacological research, academic collaboration and global team building have created an ecosystem for innovation and manufacture of mRNA-based vaccines (1). The leading role of the National Institute of Allergy and Infectious Diseases (NIAID), Applied Science, collaborations among academia, industry, and global teams have bolstered an effective ecosystem. This system forges vaccine victories and lessons to build armamentaria for pandemic prevention, to achieve a goal of improving public health, societal prosperity, and a worldwide wellbeing (2–10).

Here we detail three vaccine victories and lessons for discussion at a multilevel: first, vaccine efficacy, second, viral pathogenicity, and third, developing a biologic arsenal against viral infections based on the knowledge of virus-host interaction. We align these with the progress and lessons learned in today’s precision/personalized medicine.

## Vaccine efficacy

We consider that vaccine efficacy is a key to development of the next generation vaccines. Specifically, this should build on and derive from the more than four decades of research and knowledge that have risen up in this field, in particular the AIDS vaccines, ranging from basic laboratory bench studies, animal model tests, to clinical trials. This has led to development of highly effective antiviral drugs, combined antiretroviral therapy (cART) and analytical/structured treatment interruption (ATI), which are applied to cure trials of AIDS to determine if anti-human immunodeficiency virus (HIV) drugs can eradicate HIV replication (3, 4, 6).

Such armamentaria, specifically the knowledge of how HIV infects CD4 T cells, have resulted in effective control of the AIDS pandemic (3, 4, 6–8). Since HIV, the causative pathogen of AIDS, targets immune cells central to human immunity, there has been little development of an effective vaccine eliciting host immunity against HIV. In contrast, collaboration and the knowledge of SARS-CoV-2 not targeting CD4 T-cells have resulted in successful development of COVID vaccines (3–10).

Taking into account the victories achieved with anti-COVID vaccines and resulting development of next generation mRNA-based vaccines, we here raise some questions for multiple level discussion on development of new AIDS and other vaccines:

1. What is the immune mechanism induced by COVID mRNA vaccines?
2. Is it a viral specific humoral immunity, or a dominant cellular immunity, or a dominant innate immunity? Or all of these?
3. Through which immune signaling pathways have vaccinees gained these immunities? Is via an interferon (IFN) related pathway that reacts to the mRNA of COVID vaccine or pathways that react to later translated Spike protein?
4. Are exosomes of antigen presenting cells generated in the draining tissues involved in this mRNA vaccine efficacy (11)? Answers to these questions are key to understanding why protection induced by COVID mRNA vaccines can have a short efficacy of about 6 months (12–17). This is the shortest efficacy among anti-viral vaccines licensed by the FDA. Such short efficacy, termed vaccine breakthrough, is not solely explained by development of the viral variants (12–32).

Moreover, the efficacy of COVID vaccines by recipients who are greater than 50- year-old generally need to be protected further from the most severe symptoms and death by boosted repeat vaccinations, and boosted protection by adding antiviral drugs such as Paxlovid. This is now called COVID – Paxlovid treatment (33–36).

Lessons learned from AIDS research have gone through full cycles per the COVID vaccines. All together, it has become clear that antiviral drug targeting the viral lifecycle play an important role in the best control of such viral pandemics. With these learned lessons concerning knowledge of vaccinology, we believe the NIAID Vaccine Research Center (VRC) should remain to play a cardinal role in vaccine development to prevent the inevitable next pandemic. Developing effective vaccines effectively remains the key. This is particularly pertinent to developments in this area, epitomized by a Coronavirus Vaccines Research and Development (R&D) Roadmap (CVR) in the University of Minnesota, or a SARS-CoV-2 Assessment of Viral Evolution (SAVE) program elsewhere (2, 14).

Note that the efficacy of a preventive vaccine differs from a therapeutic vaccine. We consider this one of the lessons that we have learned via the COVID mRNA vaccines. Their efficacy can prevent the disease for a period of time and alternatively treat disease by reducing severe COVID symptoms (12–17, 33–36).

We believe that the vaccine efficacy remains most important in developing next generation vaccines. Additionally, COVID mRNA vaccines are a victory of therapeutic vaccines now taking center stage, and to be applied to reduce the severity in other instances of different viral infections (37), lyme disease (38), parasitic disease (39), bacterial disease (40), allergy (41), autoimmunity (42), or cancer immunotherapy (43).

## Viral pathogenicity

We would like to emphasize that understanding of viral pathogenesis itself is crucial to determining the efficacy of a vaccine. It is generally recognized that viruses cause host damage in two ways: first, viral multiplication at the expense of host cells, even to a level to killing the host. Second, viral genotoxicities can directly act on host DNA, RNA and proteins. Such genotoxicities occur from the viral nucleic acids and proteins per se, which damage cell genomic DNA and RNA at the gene expression levels up to a degree that change a cell to the cancer cell, as in the infections of papillomavirus, SV40 virus, and cancer related hepatitis B or C viruses, etc.

Despite the fact that other pathogens can do similar damages, viral caused genotoxicities to human DNA can override other damages to the host cells, particularly the harm on the signaling pathways governing cellular homeostasis. This leads to two pertinent questions for discussion:

First, studies have revealed that SARS-CoV-2-specific memory T cells likely prove critical for long-term immune protection against COVID-19. Should therefore, the next generation of vaccines function on eliciting durable T cell immunity, specifically via CD4 T-cells that have been associated with the effective control and eradication of SARS-CoV-2 via activating other adaptive immune cells including antibody-producing B-cells (17–22)?

Second, should the next generation of vaccines affect the route of infection, such as a nasal spray COVID vaccine (44)? This can greatly increase the efficacy of a vaccine by blocking viral entry along the route of infection and spread, similar to that of effective polio vaccine.

We note that poliovirus targets the motor neurons in the spinal cord, and causes damage to motor neurons leading to paralysis. The most effective polio vaccine functions at the entry level of viral infection systematically, specially the digestive system (23, 24).

If we consider that the smallpox vaccine was made before we fully understood the human immune system, the polio vaccine was also made before we fully applied the systems vaccinology to develop the modern vaccines. The victories and lessons we learned from both vaccines are that the route of infection plays a key role in the efficacy of a vaccine. In the smallpox case, the damage occurs on the skin, the infections spread via the skin, and vaccinations are executed on the skin. Poliovirus is spread via the digestive system, and the vaccination occurs in the digestive system. COVID is spread via the respiratory route, so the vaccine efficacy can be improved by vaccination nasally, right at the start of human respiratory system.

Thus, understanding the molecular mechanisms of SARS-CoV-2 pathogenicity and COVID-19 immune pathogenesis underpin developing effective next generation antiviral vaccines as reported (45). Scientific gaps and opportunities are not limited as stated there. Specifically, development of SARS-CoV-2 mucosal vaccines needs to be built on the lessons learned from developing the influenza vaccines, where CD4 T-cells play a cardinal role (37, 45–49).

## Potential vaccine-related pathogenicity

The molecular mechanisms underpinning COVID-19 immune pathogenesis need to be considered as crucial in improving vaccine efficacy. A cautionary note has been raised by several studies reporting complications from the vaccine and this has fueled anti-vaccine sentiments. Complications of post-COVID-19 mRNA vaccine include myocarditis as potentially direct toxicity of viral nucleic acids and proteins on the human hosts (50–52). Although post-COVID-19 mRNA vaccine myocarditis occurs in rare cases, and it was determined under the FDA Emergency Use Authorizations that the benefits of using mRNA COVID-19 vaccines clearly outweigh such risks in all populations, the adverse events need to be addressed for the development of next generation vaccines. In addition, it is well known that HIV envelope protein (Env) is a causative agent that causes neuropathogenesis in AIDS (53–56).

Another potential adverse effect concerns genomic integration of vaccine RNA. This is based on the recent demonstration that SARS-CoV-2 sequences can integrate into the genome of infected cultured cells and of patients by a LINE-1 mediated retrotransposition mechanism (57). Though genomic integration of viral sequences is a rare event in virus infected cells, the possibility of vaccine RNA integration has been used by the anti-vaxxers as an argument against vaccination. Recent experiments tested whether viral RNA transfected into cells would integrate but no evidence for genomic integration was found (58). This is consistent with the fact that vaccine RNA that does not integrate into the genome, in contrast to RNA from the viral infection.

## Approaches to prevent the next pandemic

The mRNA vaccines against SARS-CoV-2 provide protection against severe symptoms of COVID. This protection decreases over months and variants of SARS-CoV-2, such as Omicron along with many mutations, evolve and escape immune protection. Paxlovid is an anti-SARS-CoV-2 compound, and an anti-COVID pill that inhibits the viral protease. Paxlovid effectively controls the viral infections in patients even those having fully and boosted COVID vaccinations (12–16, 33–36). The rapid development of Paxlovid is also built on the four decades of AIDS research, since Paxlovid is a drug consisting of two viral protease inhibitors, nirmatrelvir and ritonavir. The latter effectively inhibits the HIV protease activity (59, 60).

Current studies have revealed that SARS-CoV-2 causes dysfunctions in the myeloid cell compartment, disturbs myeloid progenitor cell function, and produces abnormal neutrophils and monocytes. The exact signaling pathway causing this change of cell development remains to be investigated (25–32).

Victories against AIDS have lessons for COVID. Likewise, victories against COVID have lessons for AIDS. Here we sum up three questions for discussion, aiming to the victories and in order to learn lessons in building armamentaria to prevent the next pandemic:

1. AIDS vaccines against envelope glycoprotein (Env) have run into the problem of variants due to the multiple mutations on Env. Is this lesson learned in developing the COVID vaccines?
2. Despite SARS-CoV-2 affecting the innate immune system and HIV affecting the adaptive immunity, both viruses cause pathogeneses by usurping host DNA function by the viral RNA/DNA that not only change the immune cell type but also the effector cell function. COVID vaccines are not primarily designed to elicit the functions of innate immune cells (15–20, 25–32), as the vaccines were made in the context of that the pathogenicity of SARS-CoV-2 was not yet determined. This is a marked contrast to the HIV vaccines.
3. What have we learned from COVID vaccines to design next AIDS vaccines? Can we retrospectively have a systems vaccinology mindset to learn from the effective polio vaccines? Specifically, it has been reported that a vaccine variant has caused a recent polio epidemic (23, 24).

In celebrating the victories and learning the lessons, we propose to add and discuss a chromatin vaccine (cVacc) as a possible new approach to prevent the next pandemic. The cVacc aims to stop the expression of viral nuclei acids, therefore to stop viral variant production (61–64). We consider that this strategy can greatly increase the efficacy of a vaccine.

**Figure 1:**
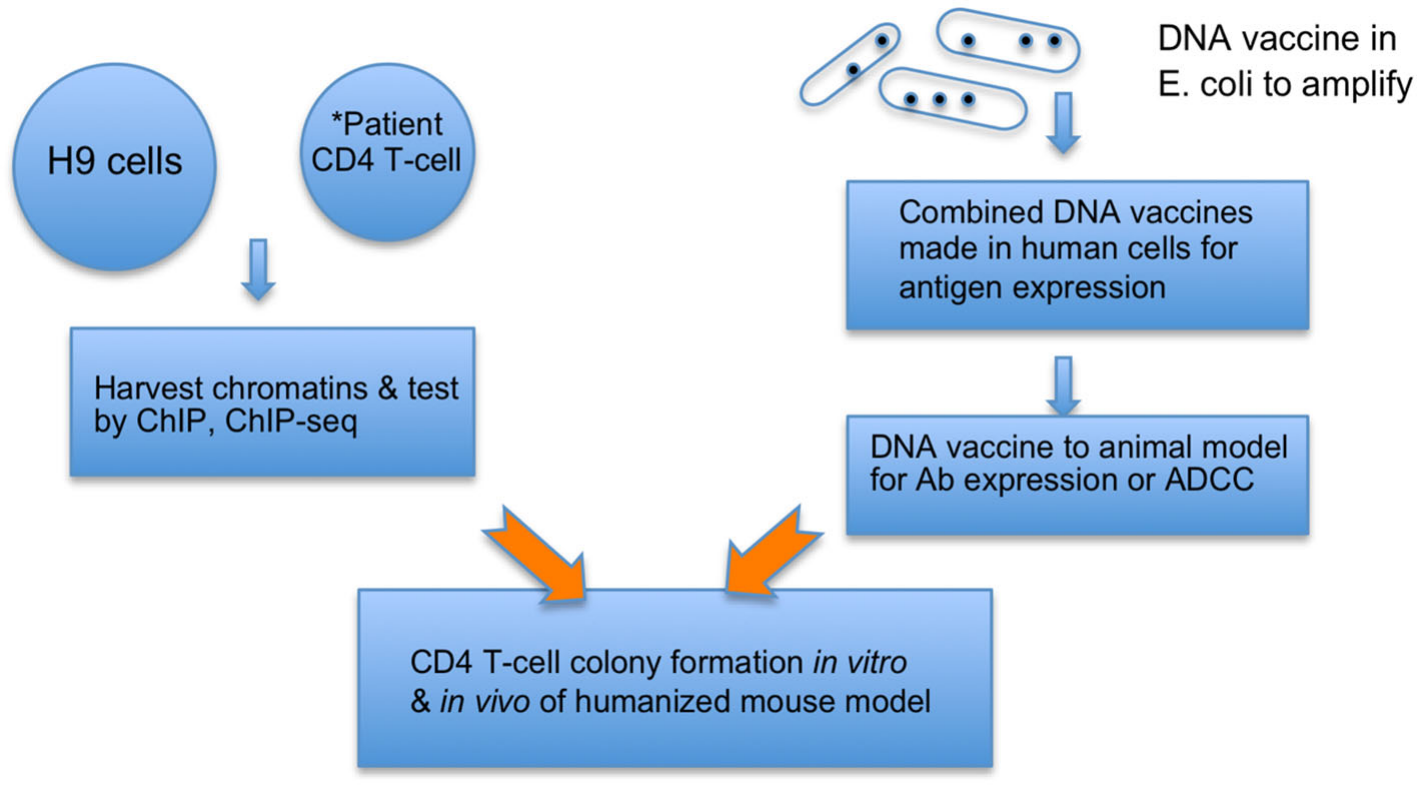
cVacc vs. a DNA vaccine. Multiple copies of provirus per a cell make H9 (ATCC CRL8543) a proper source to generate cVacc that are further verified by ChIP-exo/ChIP-seq qualitatively and quantitatively. *cVacc can be generated from patient CD4 T-cells after clonal expansion and then tested after results of H9 cVaccs, serving as autologous vaccination. The cVacc elicited immune function can be tested *in vitro* via CD4 T-cell colony formation assays, *ex-vivo* in humanized mouse models, and/or in nonhuman primate models. Note that cVacc exerts so-called binary functions: 1) to efficiently express its own immunogens, such as ncRNA and epi-markers, and 2) trigger the p21-signalosome pathway to enforce the HIV into a new human endogenous retrovirus (HERV). Patient CD4 T-cells, after *in vitro* exposure to cVacc (*in vitro* vaccination), can be infused back to accomplish an autologous or allogeneic vaccination.

The HIV cVacc elicits epigenetic immunity (63, 64). cVacc is a functional gene transcription unit with enhancer, in nucleosome format that resists nuclease degradation while mediating epigenetic silencing of viral RNA by the functions of noncoding RNA (ncRNA) and enhancer decommissioning. Furthermore, cVacc differs from the traditional vaccines in the following ways:

1. HIV cVacc consists of 2-LTR/1-LTR circles (65), triggering a signaling pathway that silences the HIV RNA transcription by decommissioning the HIV enhancer (63, 64). This vaccination quenches and prevents the genotoxicity and virulence of viral RNAs, proteins, and mutants from damaging host cells while priming CD4 T-cell differentiation to immune effector cells (**Figure 1**).
2. The production and validation of a cVacc is built on the knowledge and technology of the NIH Roadmap Epigenomics project and ENCODE consortium, specifically Omics data, which enable analyses by a systems vaccinology approach (9, 10, 61–64).
3. Further, the induced autologous vaccinated CD4 T-cells can be infused back to the patients to elicit an autologous immunization, embodying both prophylactic and therapeutic functions of the cVacc (**Figure 1**).
4. As a cVacc elicits the cell enhancer decommissioning that is the molecular mechanism governing cell differentiation and cell type, a cVacc can also act as an anticancer vaccine against cancer stem cells.

## Conclusions

Several approaches are being developed to make next generation vaccines against SARS-CoV-2 (66), and some of these could have applications against other viruses such as HIV. One approach involves making nanoparticles studded with multiple viral proteins that could generate a more potent immune response, or use of natural nano extracellular vesicles like exosomes to do this (67, 68).

The design of cVacc is built on the accomplishments and lessons gained from development of both AIDS and COVID vaccines. Therefore, cVacc embodies features of therapeutic and preventive vaccines. This aligns achievements and lessons learned in vaccine development with the advancements in now personalized/precision medicine.

We hope multilevel discussions on the two vaccines, COVID and AIDS, can forge knowledge, good laboratory practice (GLP), and research & development (R&D) to build a biologic arsenal that not only can cure the current but also prevent next pandemics.

## Author contributions

JZ: Writing – original draft, Writing – review & editing. PA: Writing – review & editing. RJ: Writing – review & editing. CC: Writing – review & editing. CC: Writing – review & editing.
